# The Innovation/Access Tradeoff, Part 1000

**DOI:** 10.1017/jme.2025.55

**Published:** 2025

**Authors:** Michael A. Carrier

**Affiliations:** Rutgers Law School, Camden, New Jersey, United States

**Keywords:** Patent thickets, Product hops

## Abstract

From the perspective of intellectual property (IP) and antitrust law, the overriding question in the pharmaceutical industry is how to navigate the tradeoff between innovation and access. It is into this debate that William Feldman steps with his important article adapted from recent testimony before the Senate Judiciary Committee. Dr. Feldman discusses an array of anticompetitive behavior. In this response piece, I focus on “patent thickets” and “product hopping” to emphasize how they often harm consumers without any innovation justification and how they can be addressed.

From the perspective of intellectual property (IP) and antitrust law, the overriding question in the pharmaceutical industry is how to navigate the tradeoff between innovation and access. In other words, how can we foster innovation in the form of potentially life-saving medicines while ensuring that as many patients as possible are able to obtain access? Drug companies deserve credit for developing important treatments. But they also have engaged in conduct that delays generic entry without any innovation-based justification.

It is into this debate that William Feldman steps with his important article adapted from recent testimony before the Senate Judiciary Committee. Dr. Feldman discusses an array of anticompetitive behavior that has been used for inhalers for asthma and chronic obstructive pulmonary disease (COPD) and GLP-1 receptor agonists treating diabetes and weight loss. Through his analysis of drug-device combinations, Feldman raises concern with late-acquired patents, device patents not connected to listings in the “Orange Book”^1^ maintained by the US Food and Drug Administration (FDA), “patent thickets,” and “product hopping.” In this response piece, I focus on these latter two activities to emphasize how they often harm consumers without any innovation justification and how they can be addressed.

## Patent thickets

The first activity is patent thickets. These are collections of patents that drug companies amass to delay competition. The thickets “are generally built from ‘secondary patents’ that take the form of minor alterations to an existing drug rather than new chemical entities.”^2^ These alterations include, for example, “changing the formulation (extended release), dosage, or route of administration (such as capsules, tablets, and topicals).”^3^

In some industries, firms need to put together collections of patents. As Sean Tu and I have explained, in the high-technology industry, there are hundreds, or even thousands, of patents in a single product.^4^ Companies in this setting accumulate patents to increase their bargaining power in licensing technology to each other.^5^

In contrast, a drug company “tends to have all of the patents it needs to enter the market.”^6^ Brand-name drug firms need not obtain a license from a generic firm to reach the market.^7^ Further casting doubt on the need for (and raising concern with) large thickets is the industry’s complex regulatory regime and market concentration.^8^

Perhaps the most famous pharmaceutical patent thicket is AbbVie’s Humira, which until recently was the world’s best-selling drug, and which treats a number of diseases including arthritis, Crohn’s disease, ulcerative colitis, and plaque psoriasis.^9^ AbbVie collected more than 130 patents on Humira, with dozens obtained shortly before the expiration of the patent on the active ingredient.^10^ In fact, over 90% of AbbVie’s patents were issued more than a decade after the drug was marketed, with the patents covering less innovative aspects of the drug.^11^

Companies facing such thickets have difficulty avoiding all of the patents. Drugs in the twentieth century were small-molecule therapies in the form of compounds produced through chemical synthesis.^12^ Generic manufacturers needed to address patents but a single drug did not tend to have many patents.^13^ In contrast, biologics are large molecules derived from living organisms and tend to be covered by more patents.^14^ Not only are they more complex, but the requirement that follow-on manufacturers offer products that are merely similar (as opposed to, for generics, equivalent) means that “there can be patents that do not claim the reference biologic product itself but are potential threats to entry for biosimilars.”^15^

## Product hopping

The second generic-delaying activity is product hopping.^16^ Steve Shadowen and I have defined the behavior to include instances in which a brand manufacturer “(1) reformulates the product in a way that makes the generic non-substitutable and (2) encourages doctors to write prescriptions for the reformulated product rather than the original.”^17^ The combination of these actions underscores the lack of innovation as the drug company does not *expand* its prescription base but just *migrates* it to the new version to block generics.

Every time the brand firm changes the drug even slightly, the generic cannot be substituted. The most effective form of competition — substitution at the pharmacy counter — requires an “AB rating,” which mandates the generic to be “therapeutically equivalent” (having the same active ingredient, form, dosage, strength, and safety/efficacy profile) and “bioequivalent” (absorbed into the body at the same rate).^18^ Each switch prevents equivalence, sending the generic back to the drawing board to reformulate the drug, obtain FDA approval, and fight a new set of patents.^19^

Product hopping not only delays generic entry but also can harm innovation as brand firms withhold advances from the market to use later as part of a product hop. Three examples illustrate. In one, the brand firm delayed seeking a new indication for the original product, reserving it for a reformulation, even though “data necessary to get the new indication was available much earlier.”^20^ In a second, the company conceded that the “principal reason … for not seeking FDA approval” for off-label uses was that it “wanted to reserve them for a later promotional campaign for its reformulated product.”^21^ And in a third, the brand firm waited until generic competition for the original drug was imminent before introducing the reformulated version even though it had obtained FDA approval three years earlier.^22^

The product hopping cases that have been litigated in the courts demonstrate the concern with the behavior. One example involves AstraZeneca’s switch from heartburn-treating Prilosec to Nexium to receive an additional 13 years of patent protection.^23^ Even though there was almost no difference between the drugs, the company aggressively promoted Nexium to doctors while stopping its promotion of Prilosec.^24^ The switch did not make economic sense since the firm stopped marketing its most profitable ($4 billion in revenues) drug, sales increased less than for other drugs in the class, and an expert told doctors they “should be embarrassed” if they prescribe the “same drug” Nexium.^25^

Another example is opioid-dependence-treating Suboxone, for which Reckitt switched from tablets to sublingual (under-the-tongue) film. The company publicly announced the removal of tablets for safety reasons (even though tablets were safer), waited 6 months to remove them, disparaged (and raised the price of) Suboxone tablets, and promoted Suboxone film to doctors.^26^ None of this made economic sense: raising the price of tablets (even though film was more expensive) was costly, as was warning of false safety concerns, all of which led to a result of “substantially reduced profit margins” on $700 million in annual sales.^27^

A third example is Alzheimer’s-treating Namenda. To obtain 14 more years of patent protection, Forest stopped actively marketing its twice-daily immediate-release (IR) version, robustly promoted the new once-daily extended release (XR), sold XR at discount, announced the discontinuance of IR, and published letters urging a switch to XR.^28^ This made no sense as Forest pulled one of its best-selling drugs ($1.5 billion in annual sales) off the market to suffer “20% franchise disruption” and the loss of “tens if not hundreds of millions of dollars.”^29^

In short, in several product hopping cases, the behavior only makes sense by harming rivals.

## Legislative solutions

Feldman supports legislation that would address these and other related issues. In this section, I focus on the legislation that the Senate Judiciary Committee considered in 2024: product hopping, citizen petitions, and agency coordination, as well as legislation on settlements and patent thickets.^30^

### Product hopping

On product hopping, S. 150, the Affordable Prescriptions for Patients Act of 2023, until recently provided an effective approach. In July 2024, the legislation, which (as discussed below) now addresses only patent thicketing, was amended to remove the product hopping provisions. That is unfortunate.

The provisions gave the FTC the power to challenge as anticompetitive two types of product hops. The first, a “hard switch,” occurs when the old drug is removed from the market. Courts have appropriately found that such switches could violate antitrust law.^31^ The second is a “soft switch,” which occurs when the old drug remains on the market. Courts have not sufficiently appreciated the harms of this conduct. For example, despite the array of questionable activity accompanying the switch from Prilosec to Nexium discussed above,^32^ the *Walgreens* court found that there was no allegation that AstraZeneca “eliminated any consumer choices” but claimed that it “added choices,” with superiority determinations “left to the marketplace.”^33^ Such statements do not recognize the unique pharmaceutical marketplace, in which buyers (consumers, insurance companies) differ from deciders (doctors), with the disconnect creating room for anticompetitive behavior.^34^

The industry and its defenders consistently complain that product hopping legislation would stifle innovation.^35^ But that is not persuasive. As Genevieve Tung and I have shown, similar innovation-based complaints trace back at least 60 years and appear every time Congress considers a legislative proposal that would (even modestly) restrict patents or apply antitrust.^36^ As former HHS Secretary Alex Azar lamented, the industry constantly recycles the “tired talking points” that “if one penny disappears” from its profit margins, “American innovation will grind to a halt.”^37^

In fact, the earlier version of S. 150 allowed a drug company to offer justifications based on showing that it had safety, supply disruption, or procompetitive reasons for the switch.^38^ The legislation also applied a deferential analysis that allowed the company to show that it would have undertaken the activity regardless of its effect on competition. In crediting all legitimate reasons (as opposed to weighing anticompetitive against procompetitive effects), the legislation applied a version of the “no economic sense” test that is as deferential as any test courts have applied in antitrust law.^39^

### Patent thickets

On patent thickets, the Affordable Prescriptions for Patients Act of 2023 mentioned above passed the Senate.^40^ The legislation limits the number of patents that can be asserted in litigation. While this offers an advantage over the current system, it would only modestly address the problem of patent thickets.

The bill would limit the biologic manufacturer to asserting 20 patents in litigation, but that cap only applies to patents that are (1) in certain categories^41^ and (2) are filed more than four years after the product’s approval or claim a manufacturing process that the biologic manufacturer does not use. The cap would not restrict other types of patents, and in any event, for administrability reasons judges typically limit the number of patents litigated.

A more direct effect would come from legislation introduced in January 2024 by Senators Welch, Braun, and Klobuchar that would allow “[p]atent holders who have created a thicket” to “assert only one patent per thicket in litigation.”^42^ The legislation also “prohibits a patent owner from asserting multiple patents from the same thicket in separate actions against the same alleged infringer to circumvent the intent of the law.”^43^ This legislation would have a more dramatic effect on thickets, as patent holders could no longer rely on the density of their thickets to swamp a rival in endless litigation but would need to choose the strongest patent to litigate.

An effective non-legislative approach to the thicket problem is to change the “terminal disclaimer” practice, as the Patent Office proposed in May 2024.^44^ Patent owners are entitled to obtain patents similar to existing ones if they agree to a terminal disclaimer that prohibits the second patent from extending the term of the first.^45^ Sean Tu, Rachel Goode, and William Feldman found that “[a]lmost half of all biologic patents involved in litigation from 2010 to 2023 had terminal disclaimers,” that “[t]hese patents spiked just as 12-year statutory exclusivity periods were ending,” and that “[t]he scale and timing of these patenting practices suggest that biologic firms may be using patents with terminal disclaimers to strengthen barriers to biosimilar entry.”^46^

The proposed rule, which was withdrawn late in 2024, would have required a patentee using a terminal disclaimer to agree that the patent is “enforceable only if the patent is not tied and has never been tied … to a patent by one or more terminal disclaimers filed to obviate nonstatutory double patenting in which … any claim has been finally held unpatentable or invalid.”^47^ The Patent Office explained that the rule “promote[s] innovation and competition by reducing the cost of separately challenging each patent in a group of multiple patents directed to indistinct variations of a single invention.”^48^

### Settlements

Legislation also targeted settlements. Brand firms have colluded with generic companies, paying them to delay entering the market. Consumers are harmed from collusion as generics delay entry from the payment as opposed to the patent. In 2013, the Supreme Court made clear that these settlements could violate antitrust law.^49^ Since then, the number of anticompetitive settlements has fallen.^50^

But legislation still is necessary. In disregard of the decision, some courts have assumed that entry before the end of the patent term is automatically procompetitive or have applied an excessively narrow interpretation of payment. For example, in *FTC v. AbbVie*, the brand firm provided the generic with a drug at a price “well below what is customary” but the court (despite recognizing the deal’s “large value”) concluded that it “was not a reverse payment.”^51^ And despite the Supreme Court’s overturning of the “scope-of-the-patent” test in ruling that antitrust law had a role to play within the patent term, the *AbbVie* court and Administrative Law Judge in the FTC’s proceedings in *Impax* assumed that entry before patent expiration was procompetitive.^52^ S. 142, the Preserve Access to Affordable Generics and Biosimilars Act, would address these issues by offering a reasonable interpretation of payment and raising the standard to presumptive illegality for settlements with payment and delayed entry.^53^

### Citizen petitions

Legislation on “citizen petitions” filed with the FDA also would be beneficial. These petitions are meant to raise legitimate concerns but have been used to delay generic entry. Carl Minniti and I found that the FDA denies 92% of “505(q)” petitions (which are filed against a pending generic), with the figure increasing to 98% for petitions filed shortly before the expiration of a patent or FDA exclusivity.^54^ The FDA has shown “concern … that section 505(q) may not be discouraging the submission of petitions that are intended primarily to delay the approval of competing drug products and do not raise valid scientific issues.”^55^ In finding that anticompetitive entry-delaying conduct can constitute sham behavior not entitled to immunity, S. 148, the Stop STALLING Act, gives the FTC needed authority to put a stamp of disapproval on abusive citizen petitions.^56^

### Interagency coordination

Other legislation, like S. 79, the Interagency Patent Coordination and Improvement Act of 2023, could increase coordination between government agencies like the FDA and US Patent and Trademark Office (PTO). Senators Hassan and Cassidy, supporters of the legislation, highlighted two examples of how the lack of coordination has contributed to patent thickets. One involves the PTO granting patents to companies that “disclosed the manufacturing process to the FDA more than a year before submitting the patent application” even though such use should prevent the issuance of a patent.^57^ A second example involves statements that manufacturers make to the FDA (e.g., “a product is the same as another one already on the market”) that “contradict statements they made to the PTO.”^58^

## Conclusion

Dr. Feldman has provided a helpful analysis of how anticompetitive conduct can delay generic entry. The legislation discussed above would not harm innovation but would have a meaningful effect for consumers.

## References

[1] The Orange Book is a collection of drugs and their associated patents. Approved Drug Products with Therapeutic Equivalence Evaluations, *44*^ *th* ^ *ed.* (Office of Generic Drug Policy, US Department of Health & Human Services, 2024), https://www.fda.gov/media/71474/download.

[2] M.A. Carrier and S.S. Tu, “Why Pharmaceutical Patent Thickets Are Unique,” Texas Intellectual Property Law Journal 32 (2024): 79, 82.

[3] *Id.*

[4] *Id.* at 84.

[5] *Id.*

[6] *Id.* at 94.

[7] *Id.*

[8] *Id.* at 98–102.

[9] *Id.* at 88; Mayor & City Council of Baltimore v. AbbVie Inc., 42 F.4th 709, 711 (7th Cir. 2022).

[10] C. Koons, “This Shield of Patents Protects the World’s Best-Selling Drug,” *Bloomberg*, September 7, 2017, https://www.bloomberg.com/news/articles/2017-09-07/this-shield-of-patents-protects-the-world-s-best-selling-drug.

[11] Carrier and Tu, *supra* note 2: 88 (patents covered the first indication (treatment of rheumatoid arthritis), formulations (such as double concentration), secondary indications (treatment of Crohn’s disease), purity levels and method of manufacture, and additional indications (like juvenile diseases)).

[12] M.A. Carrier and C.J. Minniti III, “Biologics: The New Antitrust Frontier,” University of Illinois Law Review 2018 no. 1 (2018): 1, 3.

[13] *E.g.*, J. Wu and C. Wan-Chiung Cheng, “Into the Woods: A Biologic Patent Thicket Analysis,” Chicago-Kent Journal of Intellectual Property 19, no. 1 (2020): 93, 159.

[14] Carrier and Minniti, *supra* note 12: 5.

[15] Wu and Cheng, *supra* note 13: 162.

[16] Product hopping has been more common with small molecule drugs, where the complexity and difference between product generations is less than with biologics. Carrier and Minniti, *supra* note 12, at 27–31.

[17] M.A. Carrier and S.D. Shadowen, “Product Hopping: A New Framework,” Notre Dame Law Review 92 no. 1 (2016): 167, 168.

[18] “Orange Book Preface,” Food and Drug Administration, https://www.fda.gov/drugs/development-approval-process-drugs/orange-book-preface.

[19] House Energy and Commerce Committee, (Subcommittee on Consumer Protection & Commerce) Hearing on Profits over Consumers: Exposing How Pharmaceutical Companies Game the System, 116^th^ Cong., at 1, (Sept. 19, 2019) (statement by M. A. Carrier), https://papers.ssrn.com/sol3/papers.cfm?abstract_id=3464850.

[20] Carrier and Shadowen, *supra* note 17: 202.

[21] S. Shadowen, et al., “Bringing Market Discipline to Pharmaceutical Product Reformulations,” International Review of Intellectual Property and Competition Law 42 no. 6 (2011): 698, 710. (noting that the firm “was concerned” that generics “would undermine sales” of the new drug).

[22] New York ex rel. Schneiderman v. Actavis, 787 F.3d 638, 647 (2d Cir. 2015) (henceforth referred to as *Namenda*).

[23] Walgreen v. AstraZeneca Pharmaceuticals, 534 F. Supp. 2d 146, 148-49 (D.D.C. 2008).

[24] *Id.*

[25] Carrier and Shadowen, *supra* note 17: 224.

[26] In re Suboxone Antitrust Litigation, 64 F. Supp. 3d 665, 674 (E.D. Pa. 2014).

[27] *Id.*

[28] *Namenda*, 787 F.3d at 648.

[29] Carrier and Shadowen, *supra* note 17: 228. For additional examples, see *id.*: 223–29.

[30] Feldman supports other legislation that, for space reasons, I don’t address here.

[31] *Namenda*, 787 F.3d at 655.

[32] *See supra* notes 23–25 and accompanying text.

[33] *Walgreen*, 534 F. Supp. 2d at 151.

[34] Carrier and Shadowen, *supra* note 17: 179–80.

[35] P.J. Pitts, “Drug Bill Carries Serious Side Effects, No Benefits,” *Chron*, July 11, 2019, https://www.chron.com/neighborhood/memorial/opinion/article/PETER-J-PITTS-Drug-bill-carries-serious-14079809.php.

[36] M.A. Carrier and G. Tung, “The Industry that Cries Wolf: Pharma and Innovation,” *STAT*, September 26, 2019, https://www.statnews.com/2019/09/26/innovation-boy-cried-wolf-pharma-industry/.

[37] S. Faulkner, “HHS Secretary Calls on Drug Companies To ‘Stop the Price Hikes,’” *Drug Delivery Business News*, May 16, 2018, https://www.drugdeliverybusiness.com/hhs-secretary-calls-on-drug-companies-to-stop-the-price-hikes/.

[38] S. 150 § 2(b)(3), reported to Senate Mar. 1, 2023, https://www.congress.gov/bill/118th-congress/senate-bill/150/text/rs.

[39] *Id.*

[40] All Actions: S. 150 – 118th Congress (2023-2024), https://www.congress.gov/bill/118th-congress/senate-bill/150/all-actions.

[41] *Id.* § 2(a)(2)(B) (applying cap to patents that “claim the biological product … or a use of that product … or a method or product used in the manufacture” of the product).

[42] “Bill to Address Patent Thickets,” Peter Welch, US Senator for Vermont, https://www.welch.senate.gov/wp-content/uploads/2024/01/Welch.Braun_.Klobuchar-Patent-bill.pdf (last visited July 31, 2024); “Welch, Braun, and Klobuchar Introduce Bipartisan Legislation to Streamline Drug Patent Litigation, Lower Cost of Prescription Drugs,” Peter Welch, US Senator for Vermont, Jan. 12, 2024, https://www.welch.senate.gov/welch-braun-and-klobuchar-introduce-bipartisan-legislation-to-streamline-drug-patent-litigation-lower-cost-of-prescription-drugs/.

[43] *Id.*

[44] “Proposed Changes to Terminal Disclaimer Practice To Promote Innovation and Competition,” USPTO, May 9, 2024, https://www.uspto.gov/about-us/news-updates/proposed-changes-terminal-disclaimer-practice-promote-innovation-and; *See generally* S.S. Tu, et al., “Biologic Patent Thickets and Terminal Disclaimers,” *JAMA* 331 (2024): 355 (finding that half of biologic patents had terminal disclaimers, which reflect only “trivial changes” but make it harder for biosimilars to enter the market). 10.1001/jama.2023.25389 PMC10722383 38095894

[45] Tu et al., *supra* note 44.

[46] *Id.*

[47] Terminal Disclaimer Practice To Obviate Nonstatutory Double Patenting, 89 Fed. Reg. 40439 (proposed May 10, 2024), https://www.federalregister.gov/documents/2024/05/10/2024-10166/terminal-disclaimer-practice-to-obviate-nonstatutory-double-patenting.

[48] USPTO, *supra* note 44.

[49] *FTC v. Actavis*, 570 US 136 (2013).

[50] Bureau of Competition, FTC, Agreements Filed with the Federal Trade Commission under the Medicare Prescription Drug, Improvement, and Modernization Act of 2003: Overview of Agreements Filed in FY 2017, (Federal Trade Commission, 2017), https://www.ftc.gov/system/files/documents/reports/agreements-filed-federal-trade-commission-under-medicare-prescription-drug-improvement-modernization/mma_report_fy2017.pdf.

[51] 107 F. Supp. 3d 428, 436 (E.D. Pa. 2015), *aff’d*, 976 F.3d 327 (3d Cir. 2020).

[52] *Id;* In the Matter of Impax Laboratories, Inc., No. 9373, at 144, 146 (FTC ALJ Chappell May 18, 2018).

[53] S. 142, Preserve Access to Affordable Generics and Biosimilars Act § 3(a), https://www.congress.gov/bill/118th-congress/senate-bill/142?q=%7B%22search%22%3A%22s+142%22%7D&s=1&r=1.

[54] M.A. Carrier and C.J. Minniti III, “Citizen Petitions: Long, Late-Filed, and At-Last Denied,” American University Law Review 66 (2016): 305.

[55] *Report to Congress: Eighth Annual Report on Delays in Approvals of Applications Related to Citizen Petitions and Petitions for Stay of Agency Action for Fiscal Year 2015*, (FDA, 2016): at 8.

[56] S. 148, Stop STALLING Act, https://www.congress.gov/bill/118th-congress/senate-bill/148/text.

[57] See Letter from Senators Margaret Wood Hassan & Bill Cassidy to PTO Director Kathi Vidal & FDA Administrator Dr. Robert M. Califf (May 25, 2022), https://www.cassidy.senate.gov/wp-content/uploads/media/doc/help_-_pto_fda_patent_letter_-_5_2022.pdf.

[58] *Id.*; *see also id.* (noting that contradiction “suggests the PTO examiners were unaware of the applicants’ disclosures to the FDA and puts into question the validity of these patents”).

